# Effect of Heat Treatment on the Microstructure and Mechanical Properties of 18Ni-300 Maraging Steel Produced by Additive–Subtractive Hybrid Manufacturing

**DOI:** 10.3390/ma16134749

**Published:** 2023-06-30

**Authors:** Mahmoud Osman, Sheida Sarafan, Priti Wanjara, Fabrice Bernier, Sila Ece Atabay, Javad Gholipour, Marjan Molavi-Zarandi, Josh Soost, Mathieu Brochu

**Affiliations:** 1 National Research Council Canada, Montréal, QC H3T 1J4, Canada; mahmoud.osman@cnrc-nrc.gc.ca (M.O.); sheida.sarafan@cnrc-nrc.gc.ca (S.S.); sila.atabay@nrc-cnrc.gc.ca (S.E.A.); javad.gholipourbaradari@cnrc-nrc.gc.ca (J.G.); 2 Department of Mining and Materials Engineering, McGill University, Montréal, QC H3A 0C5, Canada; mathieu.brochu@mcgill.ca; 3 National Research Council Canada, Boucherville, QC J4B 6Y4, Canada; fabrice.bernier@cnrc-nrc.gc.ca (F.B.); marjan.molavi-zarandi@cnrc-nrc.gc.ca (M.M.-Z.); 4 Matsuura Machinery USA Inc., St. Paul, MN 55102, USA; josh.soost@matsuurausa.com

**Keywords:** additive–subtractive, hybrid manufacturing, 18Ni-300 maraging steel, heat treatment, mechanical properties, microstructure, X-ray micro-computed micrography, micropore analysis

## Abstract

The present work investigates the effectiveness of two heat treatment cycles—solution treatment + aging (STA) and direct aging (DA)—on optimizing the microstructure and enhancing the mechanical properties of 18Ni-300 maraging steel (300 MS) produced by additive–subtractive hybrid manufacturing (ASHM). The STA treatment led to a fully martensitic microstructure with minor remnants of the cellular substructures associated with the solidification conditions in ASHM. DA resulted in some reverted austenite and partial dissolution of the cellular morphologies into shorter fragments. Despite the contrasting microstructures, the tensile strength and the macro- and micro-hardness were comparable between STA and DA conditions. By contrast, the potential for improving the ductility was higher with the DA heat treatment. This is attributed to the higher reverted austenite content in the samples treated by DA, i.e., up to a maximum of 13.4% compared to less than 3.0% in the STA samples. For the DA sample with the highest reverted austenite content of 13.4%, the highest local and global fracture strain values of 30.1 and 5.9 ± 0.6% were measured, while the respective values were 23.4 and 4.4 ± 0.1% for the corresponding STA sample. This work suggests that DA of 300 MS produced by ASHM is sufficient to achieve comparable hardness and tensile strength to STA, whilst maintaining reasonable ductility. Avoiding the solution treatment cycle, with its appreciably higher temperatures, could benefit the dimensional stability and surface quality that are important for ASHM of 300 MS parts.

## 1. Introduction

Grade 18Ni-300 maraging steel (hereinafter 300 MS) has been extensively studied over the past few decades for its unique mechanical properties, manifested in high tensile strength, superior hardenability, and excellent toughness [1,2]. In addition, 300 MS features good weldability and machinability along with dimensional stability during the aging heat treatment, which promotes its use in a wide range of applications within the aerospace sector, precision gear market, and tooling industry, to name a few [3,4]. The most practiced heat treatment cycle for wrought 300 MS comprises: (1) A solution treatment (ST) for austenitization, at which stage the alloy is heated above its austenitic temperature (typically between 815 and 840 °C) and then slowly cooled down in air to form a heavily dislocated, but soft martensitic matrix [5]. This is followed by (2) an aging treatment (between 480 and 500 °C for several hours) to enhance the strength through the precipitation of nanosized, hard intermetallic compounds, such as Fe_3_(Ni,Mo), FeMo, Fe_7_Mo_6_, and Ni_3_Ti, in the soft martensite matrix [6,7]. The low carbon composition (maximum 0.03 wt.%) of 300 MS distinguishes it from other types of steels and practically eliminates carbide precipitation; thus, the intermetallic precipitates during aging are the main strengthening phase in 300 MS through inhibiting the movement of dislocations [8].

Additive manufacturing (AM) technologies, such as laser powder bed fusion (LPBF), have been widely applied to 300 MS to produce tools/parts with intricate inner features owing to its favorable weldability properties [9]. The distinctly different microstructural features of 300 MS produced additively—as noticeable in the refined microstructure and the melt pool-induced meso-structures generated by LPBF processing—in contrast to wrought ones can impact their mechanical performance [7]. Additionally, subsequent heat treatment of LPBF 300 MS can elevate its ultimate tensile strength (UTS) from ~1000 to 1300 MPa in the as-built (AB) conditions to ~1900–2100 MPa in the aged conditions, but at the expense of ductility, which reduces from ~10–14% to ~3–4%, respectively [10]. Unlike wrought 300 MS, there is no commonly accepted heat treatment cycle for 300 MS produced by LPBF because of the inherent differences in the microstructure, compositional uniformity, and solute micro-segregation that may occur depending on the LPBF conditions; thus, a different treatment cycle may be required [11,12,13,14,15]. To highlight this, Table 1 was constructed to summarize the reported heat treatment cycles at peak hardness and strength from a wide range of studies. The presented data reveal the comparative effect of solution treatment + aging (STA) and direct aging (DA) treatments on the hardness and UTS of 300 MS fabricated by LPBF processing. Overall, the STA samples exhibited a homogenized microstructure with full recovery from the cellular morphologies and scan tracks, as well as the elimination of retained austenite associated with the LPBF process [16,17,18]. The DA samples featured retained cellular substructures and an increased austenite content, as a result of the martensite reversion into austenite during aging [5,10,15,19].

However, there is a noticeable discrepancy in the reported heat treatment cycles to achieve peak hardness and UTS of 300 MS produced by LPBF processing. Table 1 shows the wide range of temperatures reported for the ST step prior to aging, which is as low as 750 °C for 2 h [17] and up to 1000 °C for 1 h [7]. Mutua et al. observed the complete disappearance of scan tracks and the cellular substructures inherited from the LPBF process, and these were replaced with fine bundles of martensitic laths when the samples were solutionized at 820 °C for 1 h [16]. Additionally, this ST temperature was sufficient to eliminate the retained austenite, as determined from X-ray diffraction (XRD) measurements with Cu Kα radiation [16]. On the other hand, other studies reported that ST at 820 °C for 1 h yielded an incomplete removal of the chemical heterogeneity induced by LPBF processing and that the microstructure retained part of the scan tracks and the cellular morphologies [5,20]. Full homogenization was reportedly achieved only at 940 °C and the typical lath microstructure was observed; however, this was accompanied by recrystallization and coarsening of the newly formed grains that—when differentiated using electron backscatter diffraction (EBSD) mapping—were found to have a grain size of ~5.8 ± 0.9 μm, similar to that of wrought 300 MS [5]. By contrast, ST at 820 °C led to a fine microstructure of 1.27 ± 1.2 μm in grain size, comparable to that in the AB conditions of 0.98 ± 0.9 μm [16]. Another contradiction in the literature is about the effectiveness of DA compared to STA for LPBF-processed 300 MS. Table 1 shows favorable hardness and/or UTS for STA samples reported in [5,16], while higher hardness and/or UTS were reported in [7,14,17] for their DA counterparts.

Another challenge for 300 MS produced by LPBF processing is the ‘stair effect’ that renders the AB surface unsuitable for most intended industrial applications of this alloy. Unsurprisingly, the application of an ASHM—an integrated LPBF technology with high-speed micromachining, where the part is being built layer-by-layer by LPBF processing and then machined after each layer or a number of layers [21,22]—is of interest for the production of highly dense parts with high-quality surface finishes on the same build envelope. This hybrid technology has been studied for different ferrous alloys [23,24,25,26,27], with a slightly higher research focus on 300 MS, both in terms of optimizing the LPBF process parameters [16,28,29] and the surface finish from machining operations [26,30]. However, to our knowledge, the heat treatment of 300 MS produced by ASHM has barely been investigated. Du et al. [28] disclosed the effect of a DA process at 500 °C for 3 h on the hardness of 300 MS produced by ASHM, and reported a hardness of 56.2 HRC. Their investigations did not consider the STA process or extend the analysis to other mechanical properties (beyond hardness) and/or microstructural characteristics. Additionally, recently, a relatively sizable/robust process window for ASHM of 300 MS to a high density (>99%) and with high-quality surface finishes (roughness ~0.32–0.80 µm) was identified [27]; however, the influence of heat treatment—after ASHM within this process window—on the mechanical performance remains outstanding for advancing a more complete process–structure–property understanding for 300 MS parts produced by ASHM.

**Table 1:** Reported heat treatment schedule at peak hardness and UTS for wrought, LPBF (standalone), and ASHM 300 MS.

Heat Treatment Conditions at Peak Properties	Process	Hardness	UTS (MPa)	El. (%)	Ref.
STA (816 °C for 0.5 h + aging 482 °C for 3 h)	Wrought	-	1944	7	[29]
STA (820 °C for 1 h + aging 490 °C for 6 h)		656 HV_0.5_	2102	2	[10]
STA (820 °C for 1 h + aging 500 °C for 6 h)			1906	-	[5]
STA (750 °C for 2 h + aging 450 °C for 6 h) DA (450 °C for 6 h)	LPBF	595 HV_0.1_ 598 HV_0.1_	1812 2060	1.7 2.6	[17]
STA (820 °C for 1 h + aging 460 °C for 5 h) DA (460 °C for 5 h)		618 HV_2_ 609 HV_2_	2033 -	5.27 -	[16]
STA (820 °C for 1 h + aging 500 °C for 6 h) DA (500 °C for 6 h)		- -	1970 1858	- -	[5]
STA (840 °C for 2 h + aging 490 °C for 2 h) DA (490 °C for 2 h)		543 HV_0.5_ 523 HV_0.5_	1790 1820	3.5 2	[18]
STA (840 °C for 1 h + aging 490 °C for 6 h) DA (490 °C for 6 h)		53.5 HRC 54.6 HRC	1943 2014	5.6 3.3	[14]
STA (900 °C for 1 h + aging 520 °C for 6 h) DA (520 °C for 6 h)		665 HV_0.2_ 654 HV_0.2_	2080 2126	5.3 6.5	[31]
STA (1000 °C for 1 h + aging 480 °C for 6 h) DA (480 °C for 6 h)		- -	1996 2021	1.7 3.7	[7]
DA (500 °C for 3 h)	ASHM	56.2 HRC	-	-	[28]

Thus, the main motivation for the present study was to further the understanding of the influence/effectiveness of the STA and DA heat treatments on 300 MS produced by ASHM. This was studied in the present work by comparing the effects of the conventional STA and DA heat treatment cycles on the obtained microstructure, phase constituents, macro- and micro-hardness, and tensile properties of 300 MS produced by ASHM over a process window that was previously identified (by the current authors) to yield high-density (>99%) parts, namely at three laser power levels (240 W, 320 W, and 380 W).

## 2. Experimental Procedure

### 2.1. Powder Characterization

The 300 MS powder used in the present work was supplied by Matsuura (St. Paul, MN, USA) and had a chemical composition as presented in Table 2. The powder used in this work was a mixture of virgin and multi-reused powder batches.

**Figure 1:**
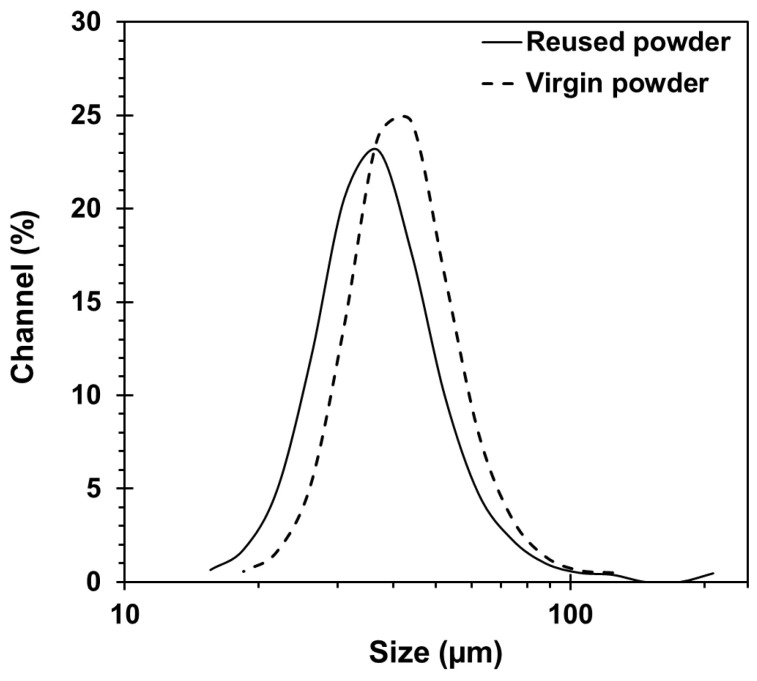
Particle size distribution of the virgin and reused 300 MS powders used in the present study.

**Figure 2:**
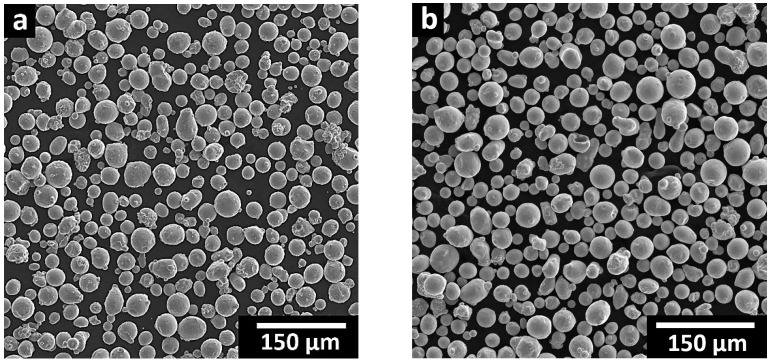
SEM micrograph of the 300 MS powder morphology: (**a**) virgin and (**b**) reused powders.

The particle size distribution of the virgin and the reused 300 MS powders was measured using a Horiba laser particle size analyzer LA-920 (Kyoto, Japan). Figure 1 shows an almost identical particle size for the virgin and reused powders, with D10, D50, and D90 of 27, 38, and 56 μm for the virgin and 24, 34, and 52 μm for the reused powders. The morphology of the virgin (Figure 2a) and reused (Figure 2b) powders was analyzed with a Hitachi SU3500 (Fukuoka-shi, Fukuoka, Japan) scanning electron microscope (SEM). The SEM micrographs revealed that the reuse of 300 MS powder had an insignificant effect on its morphology, which is consistent with earlier findings by Sun et al. [15]. Overall, both powders had predominantly spherical particles with the presence of minor powder agglomerates in the reused powder from the previous builds.

**Figure 3:**
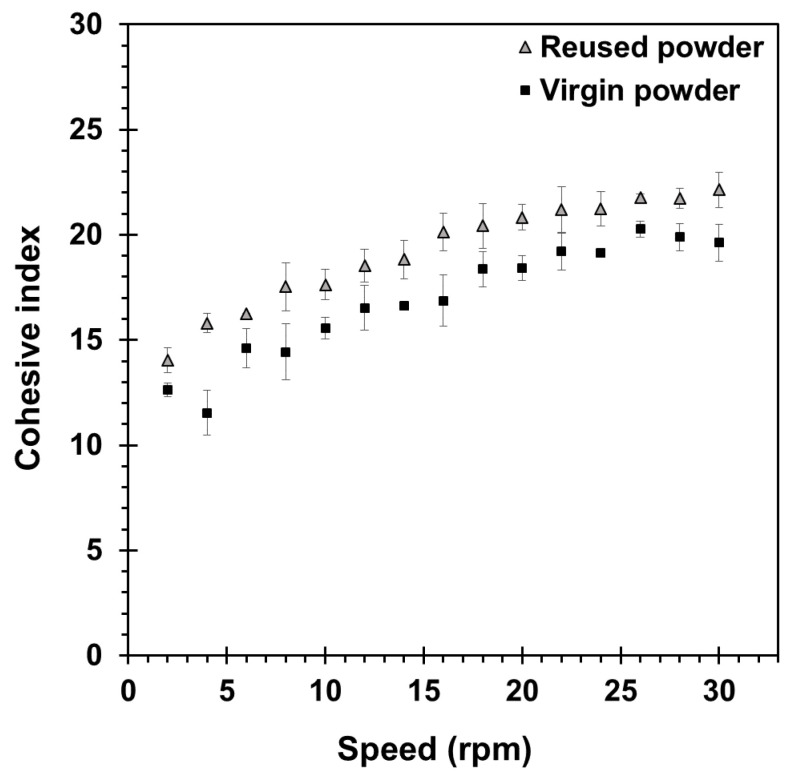
The cohesive index of the virgin and reused 300 MS powders.

The powder cohesion was evaluated using a GranuDrum^®^ from GranuTools^TM^ (Awans, Belgium). In the present study, the cohesive index was measured at drum angular velocities ranging from 2 to 30 rpm, where 40 powder images were captured at an interval of 1 frame/s at each angular velocity. The images taken were analyzed by the built-in GranuDrum^®^ software version 9.22.4.19. Figure 3 presents the cohesive index for the virgin and reused 300 MS powders used in the present study. Both powder lots exhibited relatively low cohesive index values (less than 24), which dictates good powder flowability and a homogenous spreading of the powder layer in the LPBF process, as reported earlier in [32]. It can be noted that the cohesive index response of the reused powder shifted slightly upwards compared to the virgin powder, which can be attributed to minor moisture exposure during powder handling and resulted in a relative flowability reduction [33,34,35]. Nonetheless, based on the overall similar powder characteristics (i.e., size, morphology, and flowability), the use of a mixture of virgin and multi-reused 300 MS powder for LPBF processing represents a sustainable manufacturing approach that is widely applied in practice. Recently, Sun et al. [15] demonstrated that the lifecycle of reused 300 MS powder could be prolonged to 113 times without observable degradations in the AB microstructure and mechanical properties.

### 2.2. Sample Fabrication

Three rectangular prismatic-shaped coupons of 75 × 25 × 25 mm^3^ in size were built on a 4140-steel build plate from the 300 MS powder blend (virgin + reused) using an ASHM system, specifically a Matsuura LUMEX Avance-25 system that consists of an integrated LPBF technology with high-speed micromachining. ASHM was performed under nitrogen gas atmosphere and the temperature of the build plate was kept at 50 °C during the process. Three different laser powers were studied (one prismatic coupon for each): 240 W, 320 W, and 380 W, with a unidirectional laser scanning strategy and 90° rotation between each layer. The other process parameters were selected as follows: a hatch distance of 120 μm, a layer thickness of 50 μm, a laser spot diameter of 200 μm, and scanning speeds of 700 mm/s and 1400 mm/s for the infill and contour passes, respectively. Milling was performed every 10 layers at a 2000 mm/min feed rate and a 0.1 mm depth of cut for the vertical sidewalls. After completing hybrid printing of the rectangular blocks, a 0.4 mm-thick layer was machined from the horizontal top surfaces of the blocks.

The coupons were then cut from the build plate with electrical discharge machining using a FANUC Robocut C400iB (Oshino-muram Yamanashi, Japan) system with a 0.2 mm-diameter brass wire. The wire was positioned at a height of 0.2 mm from the base plate; thus, the base of the coupons was at a height of ~0.5 mm, representing the first 10 deposited layers.

### 2.3. Heat Treatments

**Figure 4:**
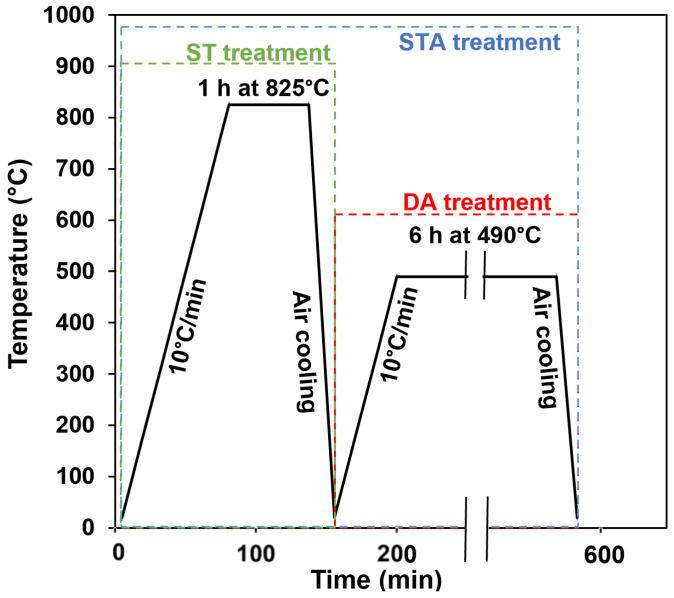
ST, STA, and DA thermal cycles.

The AB 300 MS coupons produced at laser powers of 240 W, 320 W, and 380 W were heat-treated in a Vulcan 3-550 (York, PA, USA) furnace. Two heat treatment cycles of STA and DA (aimed at peak hardness) were studied in the present work. The details of the heat treatment cycles are presented in Figure 4. The STA cycle consisted of ST at 825 °C for 1 h, followed by air-cooling to room temperature, and an aging cycle of 490 °C for 6 h, then air-cooling. The DA cycle was performed at 490 °C for 6 h, followed by air-cooling to room temperature. To avoid surface reactions during these heat treatments, the coupons were placed in envelopes made of ultra-thin stainless-steel heat-treating foil and intentionally packed with thin carburizing/paper strips; during heating, this inner layer of paper partially burned and consumed any remaining oxygen within the envelope, thereby adequately protecting the coupons from oxidation [36]. The temperature was recorded using two thermocouples, one attached to the coupon inside the stainless-steel envelope, and one located in the center of the furnace. The resulting conditions and designations assigned to the samples, prepared for characterization and testing (as described in the next section), are presented in Table 3.

### 2.4. Materials Characterization and Testing

#### 2.4.1. Microporosity Measurements

X-ray micro-computed tomography (µCT) was used to analyze 12 datasets to study the porosity evolution after the STA and DA heat treatments. These 12 datasets represent 6 samples, which were each scanned twice: before and after heat treatment. Thus, the six AB samples were fabricated under the three laser powers (240 W, 320 W, and 380 W) and then heat-treated to the STA or the DA condition. X-ray μCT was undertaken on these samples using a Nikon HMXST 225 system (Brighton, MI, USA) equipped with a Perkin-Elmer 1621AN CsI (2000 × 2000 pixels, 40 × 40 cm^2^ and 200 µm/pixel) detector panel. The X-ray μCT system was operated at a voltage of 135 kV, a current of 64 μA with a 0.25 mm Ag filter, and an exposure time of 1415 ms. Four frames per projection were taken and a voxel size of 2.7 µm was used. The volume of the inspected region was ~75 mm^3^, while the volume of the analysis region was 20 mm^3^. For the inspection, pores were filtered at 5 voxels (i.e., pores containing less than 6 voxels were excluded from the analysis). For image analysis, Dragonfly software was utilized for the 3D reconstruction to analyze the volume and size distribution of the pores. The porosity levels were detected using a lower Ostu threshold [37,38,39] to select the region of interest (ROI) of the pores.

#### 2.4.2. Microstructural Characterization

Microstructural characterization was carried out on the AB and heat-treated samples. The samples were prepared for microscopic examination by cold mounting, followed by grinding with successively finer silicon carbide (SiC) papers of 220- and 800-grit, and then by rough polishing using 9, 3, and 1 μm diamond suspensions with an alcohol-based lubricant on silk polishing cloths. Final polishing was conducted using 0.05 μm colloidal silica on a porous pad. Subsequently, the samples were chemically etched by immersing in a 2% Nital solution for 2 min. The microstructure was then observed using a laser scanning confocal/optical microscope (Keyence VK-X250, Osaka, Japan) and a Hitachi SU3500 SEM.

XRD was used to quantify the amount of retained austenite in the AB and heat-treated conditions. Diffraction patterns were recorded for the different sample conditions on a D8 Discover diffractometer (Bruker, Karlsruhe, Germany) with a Co-Kα anode (wavelength, λ = 0.178897 nm), operating at a voltage of 35 kV and a current of 45 mA. The scans were acquired over a diffraction angle (2θ) range from 20° to 110° at a scan step of 0.005°. Processing of the diffraction patterns to differentiate between the austenite (COD ID #7204807) and martensite (COD ID #1100108) peaks was performed with DIFFRAC.EVA version 4.3 software, and the direct comparison method [40] was used to quantify the austenite content in each sample.

#### 2.4.3. Mechanical Testing

The macro-hardness of the AB and heat-treated samples was measured at room temperature by Rockwell C hardness testing, in accordance with the ASTM E18 standard [41]. The reported value for each sample condition is the average of 10 measurements. The microhardness of the samples was measured at room temperature using a Clark CM-100 AT Vickers Microhardness Indenter (Sun-Tec, Novi, MI, USA), according to ASTM E384 standard specifications [42]. A 500 g load was applied for a dwell time of 15 s, and the average Vickers hardness values were measured from 10 indents for each sample condition.

For tensile testing, according to the guiding principles of the ASTM E8M standard [43], dog-bone-shaped samples with a cross-section of 6 × 3 mm^2^ and a gauge length of 25 mm were machined from the three (240 W, 320 W, and 380 W) fabricated prismatic coupons (75 × 25 × 10 mm^3^) along the build direction (BD). Tensile testing was carried out on the 240 W, 320 W, and 380 W samples in the STA and DA conditions. The tensile tests were performed at room temperature using a 250 kN testing frame integrated with a laser extensometer (MTS Systems Corporation, Eden Prairie, MN, USA) and a non-contact optical 3D deformation measurement system—often referred to as digital image correlation (DIC) (Aramis^®^, GOM-Trillion Quality Systems, King of Prussia, PA, USA). Before testing, one side of the sample was marked with two pieces of retro-reflective tape to define the gage length for the laser extensometer measurements during tensile loading, as shown in [44]. On the opposite side, the sample surface was first painted with a white background, followed by the application of a high-contrast, random pattern of black speckles, as illustrated in [45]. The accuracy of the Aramis^®^ DIC system is sensitive to the quality of this speckle pattern, and pattern recognition was verified before tensile testing to ensure proper strain recording along the entire gauge section. Tensile tests were then conducted at a constant crosshead speed of 0.4 mm/min that ensured quasi-static loading at a strain rate in the order of 10^−4^ s^−1^. Upon completion of each test, the load data, collected from the load cell of the tensile testing system, were used to calculate the engineering stress, while the strain was measured from the laser extensometer data. The average tensile properties—0.2% offset yield strength (YS), UTS, fracture strain, and elastic modulus (E)—were evaluated from the engineering stress–strain curves based on three replicates for each condition. Additionally, the Aramis^®^ DIC strain distribution maps were used to examine the strain localization behavior of the samples during tensile loading. After tensile rupture, the resulting fracture surfaces of the samples were examined with a SEM to identify the failure mechanisms associated with the DA and STA heat treatments.

## 3. Results

### 3.1. Microporosity Characterization

**Figure 5:**
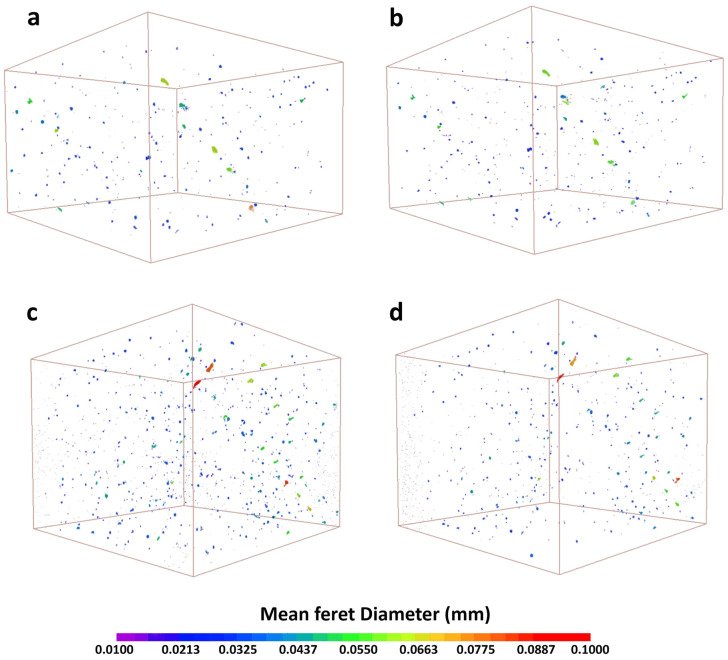
The 3D representation of the pore equivalent diameter and distribution in (**a**) AB-320W1, (**b**) STA-320W1, (**c**) AB-320W2, and (**d**) DA-320W2.

Figure 5a,b show a 3D representation of the porosity size and distribution in AB-320W1 and STA-320W1 that reveals the similar pore morphology and distribution after the STA process. It is noteworthy that only one representative STA sample condition is presented in Figure 5 for visualization, since all the other samples (240 W and 380 W) had similar results, as indicated in Table 4. The observed defects within the samples were found to be uniformly distributed and could be classified mainly into two types of porosity according to their size and morphology: gas pores and lack of fusion. The vast majority of the pores were observed to be isolated and exhibited characteristics typical of gas-induced porosity, featuring a relatively small size of less than ~40 µm and having a nearly spherical shape. These micropores likely arose from entrapped gas bubbles in the original powder and/or the produced part due to the inert argon/nitrogen atmosphere used in the gas atomization process and the LPBF manufacturing process, respectively [46]. By contrast, the observed lack of fusion in the samples was less frequent, and these pores exhibited irregular shapes with relatively large sizes above 40 µm. Their presence is typically attributed to insufficient melting among adjacent tracks and layers, while being LPBF-processed [47]. Quantitative analysis showed a similar porosity content in both the AB and STA conditions. The total number of defects was 386 in AB-320W1 and constituted a volume fraction of 0.005%, while STA-320W1 showed 425 defects with an equivalent 0.005% volume fraction of pores.

**Figure 6:**
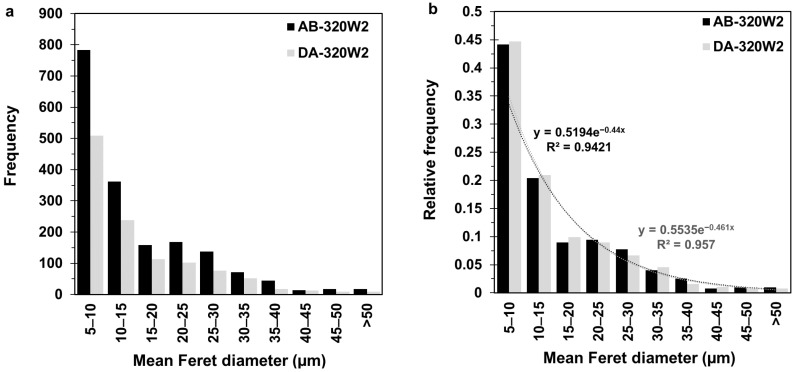
Histogram of the defect frequency (**a**) and relative frequency (**b**) size distribution in AB-320W2 and DA-320W2 samples.

Figure 5c,d present the µCT observations of the AB-320W2 and DA-320W2 samples (that also are representative of the other laser power conditions). Relative to AB-320W1, AB-320W2 showed a higher number of defects (1772) and a higher (0.016%) volume fraction of pores. It is noteworthy, however, that this observed difference in the volume fraction of pores between these as-built samples fell within the standard deviation of the bulk density previously reported by the authors in [27]. After DA of AB-320W2, DA-320W2 showed a decrease in the number of defects to 1182 pores and a lower volume fraction of 0.01% pores. For a more detailed analysis, Figure 6a presents the defect frequency size distribution of the AB-320W2 and DA-320W2 samples according to the µCT data presented in Figure 5c,d. This quantitative analysis revealed that the reduction in the defect frequency after DA occurred systematically for all defect size ranges. However, when inspected further with the relative defect frequency histogram, presented in Figure 6b, the relative frequency remained unchanged after DA. Even so, both histograms revealed an exponential decrease in the relative frequency of the defect with the defect size for both the AB and DA conditions. From these results, the calculated average defect size was 15.6 and 15.3 µm in the AB-320W2 and DA-320W2 samples, respectively. Thus, the overall changes in both the average pore size and porosity content between the AB and DA samples were negligible.

Recently, Song et al. [47] also studied DA of LPBF-processed 300 MS at 500 °C for 6 h, followed by furnace cooling. They reported a minor (0.1%) increase in the relative density (i.e., decrease in porosity) after DA, but their quantitative analysis using µCT showed that the volume fraction of defects in their AB (0.0182%) sample slightly increased after DA (0.0246%). Such contradictory findings may be related to sampling discrepancies in correlative imaging, which were addressed in the current study by µCT scanning the exact same ROI in the sample before (AB) and after heat treatment (STA or DA). Specifically, an intentional notch on each AB sample was placed using a fiducial marker as a point of reference during µCT scanning to help align and locate the exact same pores when re-examining the ROI in the samples after heat treatment (STA or DA). In this way, the datasets of the pore size distribution were correlated for not only the 320 W sample (Figure 5), but also the other laser power-processed samples (Figure 6). Table 4 presents the dataset for the 240 W, 320 W, and 380 W samples before and after heat treatment (STA and DA), which shows similar trends in the number of detected pores, average pore size, and overall percent porosity. For the 380 W samples, minor changes in the number of detected pores were noticed, as evident in Figure 5; however, the resulting percent porosity remained too low to have a significant effect on the mechanical properties of heat-treated 300 MS. Overall, it can be concluded that neither the STA nor the DA heat treatments had any significant effect on the porosity of 300 MS produced by ASHM.

### 3.2. Microstructure Characterization

#### 3.2.1. As-Built (AB) Conditions

**Figure 7:**
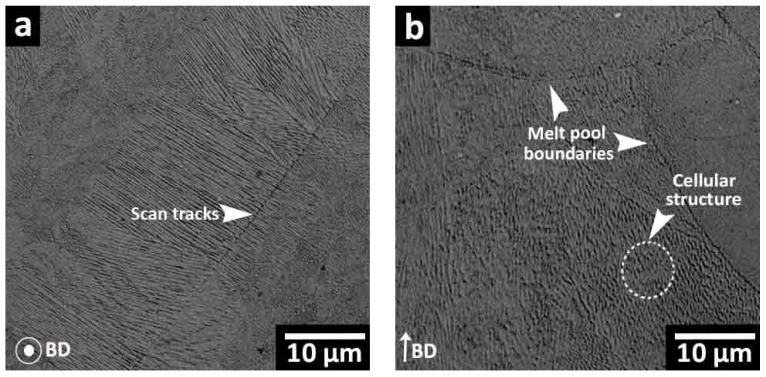
Optical microscope images showing the microstructure of the AB sample produced with a 320 W laser power: (**a**) perpendicular to the BD and (**b**) parallel to the BD.

Figure 7 shows the microstructure of the AB-320 W sample, which was discussed in detail in a previous work [27] by the authors. Additionally, this microstructure is representative of the other sample conditions, as there were no microstructural differences after ASHM at the two other laser powers (240 W and 380 W). The main features of the 300 MS fabricated by ASHM are the semi-elliptical melt pool boundaries, the overlapping scanned tracks, as well as fine (submicron-sized) cellular substructures, which are highlighted in Figure 7. These observations are aligned with previously reported microstructures for LPBF-processed 300 MS with the characteristic presence of fine cellular substructures. These are due to the solidification conditions in LPBF processing, including the high thermal gradients and solidification front velocity, in combination with the rapid cooling rate, which is of the order of ~10^6^ °C/s [16,18,31]. The AB microstructure presented here also agrees well with the previous findings of Du et al. [28] on the microstructure of 300 MS produced by ASHM.

#### 3.2.2. Solution-Treated + Aged (STA) Condition

**Figure 8:**
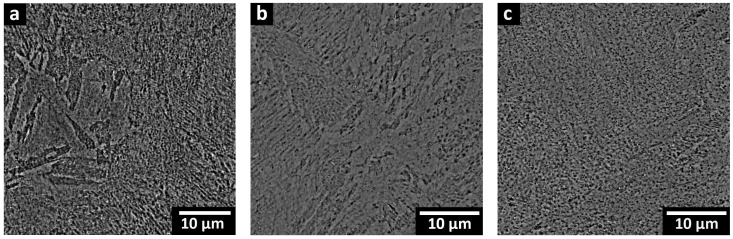
SEM micrographs of the STA microstructure processed under different laser powers: (**a**) STA-240 W, (**b**) STA-320 W, and (**c**) STA-380 W.

Figure 8a–c show the microstructure of the STA samples, where the cellular substructures induced by the LPBF process were partially retained, while the melt pool boundaries and scan tracks disappeared. Thus, ST at 825 °C for 1 h used in the present work was insufficient to completely remove the cellular substructures, and the observed traces of the cellular morphology remained in the microstructure prior to aging. Previous studies of Kucerova et al. [5] and Conde et al. [20] reported retained weld tracks and cellular features of the AB conditions after ST at 820 °C for 1 h. Then again, Mutua et al. [16] observed that ST at 820 °C for 1 h led to the complete disappearance of the scan tracks and solidification traces, as well as the replacement of the cellular morphologies with fine bundles of parallel martensitic laths. A few attempts [5,31] were reported to find the threshold ST temperature for a full recovery from the remnants of LPBF features, which involved a wide range of tested ST temperatures and a characterization of the resulting microstructure. The lowest threshold ST temperature was achieved by Bai et al. [31], who tested LPBF-processed 300 MS with a number of ST temperatures (780, 840, 900, 960, and 1020 °C) for 1 h and concluded that a ST temperature of 840 °C was sufficient to completely remove the melt track boundaries and the cellular substructures of the AB conditions.

#### 3.2.3. Direct Aging (DA) Condition

**Figure 9:**
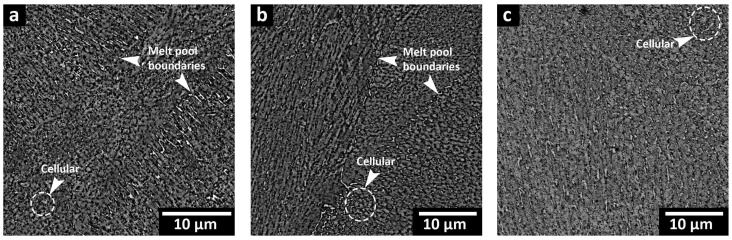
SEM micrographs of the direct-aged (DA) microstructure processed under different laser powers: (**a**) DA-240 W, (**b**) DA-320 W, and (**c**) DA-380 W. Remnants of the cellular and columnar substructures are highlighted.

The microstructure of the DA samples, as illustrated in Figure 9a–c, showed similar solidification characteristics to the AB sample. Notably, the cellular structures and molten pool boundaries were still retained after aging. The cellular walls appeared thinner and broken into discontinuous fragments for all the DA samples, regardless of the laser processing power. Image analysis revealed ~2, 4, and 5% of retained cellular walls in the 240 W, 320 W, and 380 W samples, respectively. The dissolution of the segregated alloying elements depends upon the aging temperature and holding times [31]. Some studies have reported retained cellular substructures after direct aging for 6 h at 480 °C [7] and 500 °C [47], while others reported partial disappearance at 520 °C for 6 h [31].

### 3.3. Phase Analysis

**Figure 10:**
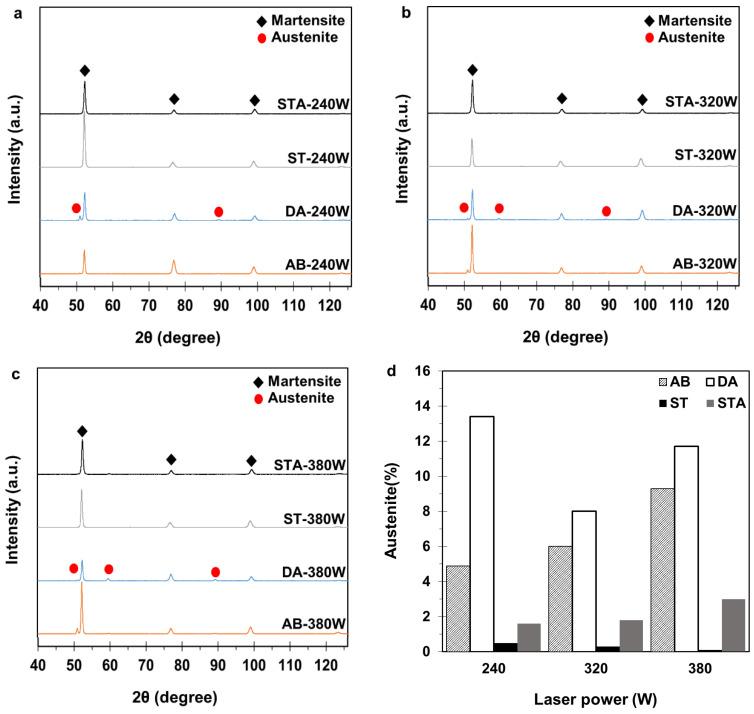
XRD patterns of 300 MS in AB, DA, ST, and STA conditions for different laser powers: (**a**) 240 W, (**b**) 320 W, and (**c**) 380 W, and (**d**) austenite content calculated from XRD data.

Figure 10a–c depict the XRD patterns of the 300 MS samples in the AB and heat-treated conditions for the three different laser powers. All the AB samples showed a small austenite (γ) peak of γ(111), which discerns the presence of retained austenite after the ASHM process, regardless of the applied laser power. This peak was eliminated in the ST samples, which confirmed the formation of a fully martensitic microstructure upon ST. Similarly, the STA samples did not show austenite peaks, confirming that almost no austenite reversion occurred on subsequent aging of the ST samples. By contrast, all the DA samples exhibited intensified γ peaks compared to the AB samples. Figure 10a shows an increase in the intensity of the γ(111) peak in DA-240 W compared to AB-240 W, which suggests the reverse transformation of martensite to austenite upon the DA treatment after ASHM.

Figure 10d shows the quantitative measurements of the austenite content retained in the AB and heat-treated samples based on the XRD data. It can be observed that the amount of retained austenite in the AB conditions increased with increasing laser power (i.e., the energy density). A retained austenite content of 4.9, 6, and 9.3% was measured for AB-240 W, AB-320 W, and AB-380 W, respectively. The ST cycle at 825 °C for 1 h eliminated the austenite retained in the AB conditions; thus, subsequent aging of the STA process resulted in a fully/predominately martensitic microstructure with a small possibility of reverted austenite in minor amounts of less than ~3% (at the detection limits of XRD).

For the DA samples, the austenite content increased, relative to the AB state, to 13.4, 8, and 11.7% for DA-240 W, DA-320 W, and DA-380 W, respectively. These findings are aligned with previous studies on LPBF-processed 300 MS that have reported increases in the austenite content after DA, such as a gradual increase in the austenite fraction with increasing DA temperature and a sharp increase in the austenite fraction when the aging temperature reached 560 °C [16,18,31]. In this latter case, the austenite content increased from 6.2% in the AB condition to 6.9% when aged at 400 °C for 6 h, but increased to 17.9% when aged at 560 °C for 6 h [31].

### 3.4. Macro- and Micro-Hardness

**Figure 11:**
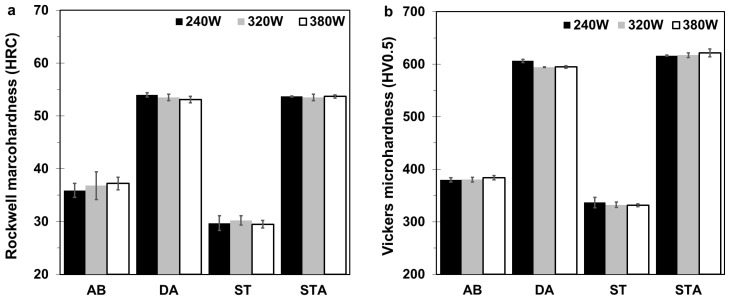
Comparison of the (**a**) Rockwell macro-hardness and (**b**) and Vickers microhardness of the AB and heat-treated samples for the different laser powers studied.

Figure 11a presents the Rockwell C hardness of the AB and heat-treated 300 MS produced by ASHM. The AB samples exhibited a hardness of 36 ± 1, 37 ± 3, and 37 ± 1 HRC, for the 240 W, 320 W, and 380 W laser power conditions, respectively. This hardness is aligned with the reported value of 35 ± 1 HRC by Chadha et al. [13] for LPBF-processed 300 MS. The hardness of the AB samples also outperformed the reported hardness of 30–32 HRC for wrought 300 MS [29]. After ST, the hardness of the ST samples dropped to 30 ± 1 HRC, for all the laser powers studied. By contrast, after aging, the STA samples exhibited a substantial increase in hardness to 54 ± 0 HRC, for all the laser powers. DA samples also showed a comparable hardness of 54 ± 1 HRC to the STA samples. These results are aligned with previous findings of Tan et al. for DA of LPBF-processed 300 MS that was reported to have a hardness in the range of 51 to 55 HRC [14].

Figure 11b illustrates the effect of the different heat treatments on the microhardness of 300 MS produced by ASHM. All the AB samples attained a comparable microhardness, regardless of the applied laser power. Specifically, the microhardness was 380 ± 4, 380 ± 5, and 384 ± 4 HV for the 240 W, 320 W, and 380 W samples, respectively. These values exceed the most commonly reported AB microhardness of 340 HV for LPBF-processed 300 MS [16,17,18], but are aligned with the reported value of 380 HV by Bai et al. [31]. After ST, the microhardness slightly dropped to 337 ± 10, 332 ± 5, and 331 ± 3 HV for the 240 W, 320 W, and 380 W samples, respectively. Despite the reduction in microhardness associated with ST, their subsequent aging (STA) was seen to tremendously improve the microhardness to 618 ± 7 HV. Similarly, the DA samples featured a comparable microhardness of 606 ± 3 HV, for all the laser powers studied. This achieved microhardness is also higher than the reported value of 594 HV for conventionally age-hardened 300 MS [11].

The current macro- and micro-hardness results revealed that the application of the ST cycle preceding the aging treatment had negligible-to-no influence on the final hardness properties of 300 MS produced by ASHM over the relatively wide operational window for laser powers (240 W, 320 W, and 380 W) associated with near-full-density parts. This finding has important significance for developing/adapting thermal cycles that are efficient for performance enhancement/optimization, whilst reigning in costs and heat treatment turnaround times, especially vis-à-vis parts produced by ASHM that have net-shape geometries and features that may be affected dimensionally when exposed to elevated temperatures and/or prolonged times.

### 3.5. Tensile Properties

**Figure 12:**
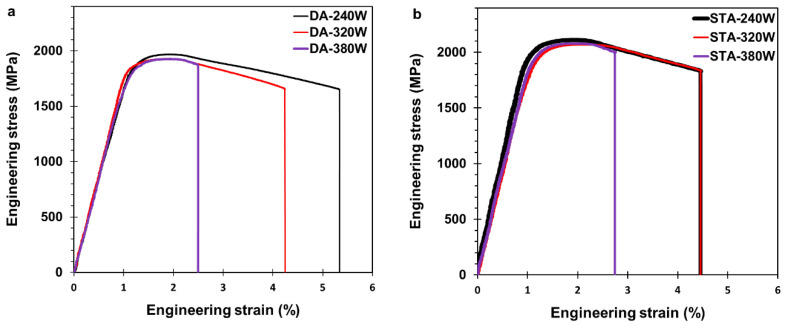
The engineering stress–strain curves of the (**a**) DA and (**b**) STA 300 MS processed by ASHM at 240 W, 320 W, and 380 W laser powers (one sample is presented per condition for better visualization).

Figure 12 shows the representative engineering stress–strain curves under tensile loading of the 240 W, 320 W, and 380 W samples in the STA and DA conditions, and their average mechanical properties are presented in Table 5, alongside our previously reported data for 300 MS in the AB state [27]. For each of the laser power conditions studied, the STA heat treatment proved to be effective in substantially increasing the strength of 300 MS produced by ASHM, with a ~100% increase in the YS and ~80% in the UTS compared to the AB state. For instance, the YS and UTS increased from 1006 and 1158 MPa in AB-240 W to 2030 and 2080 MPa in STA-240 W. Similarly, a significant increase in the strength occurred after the DA heat treatment, with the YS and UTS increasing to 1884 and 1950 MPa in DA-240 W, respectively. On the other hand, the global fracture strain significantly dropped from 12.8 ± 0.7% in AB-240 W to 4.4 and 5.9% in STA-240 W and DA-240 W, respectively. By contrast, the stiffness of 300 MS produced by ASHM remained mostly unaffected by the heat treatment, with only the STA samples showing a marginal increase in the average E value, by ~12%.

The tensile response of 300 MS in the STA and DA conditions was mostly comparable for all the laser powers studied, as evident in Figure 12 by the similar trend in the stress–strain curves that showed some relaxation upon reaching the UTS. The average UTS in the STA condition was 2073 ± 13 MPa, and it was 1939 ± 19 MPa in the DA condition. Thus, the UTS of the STA samples was slightly higher (by 4–7%) than the DA samples. For instance, STA-240 W exhibited a UTS of 2080 MPa compared to 1950 MPa for DA-240 W, at 95% CI. On the whole, however, these UTS values for the STA and DA samples agree well with reported data in previous heat treatment studies on LPBF-processed 300 MS, which ranged from 1800 to 2100 MPa at the authors’ optimum heat treatment conditions [3,11,13,16,18,31].

**Figure 13:**
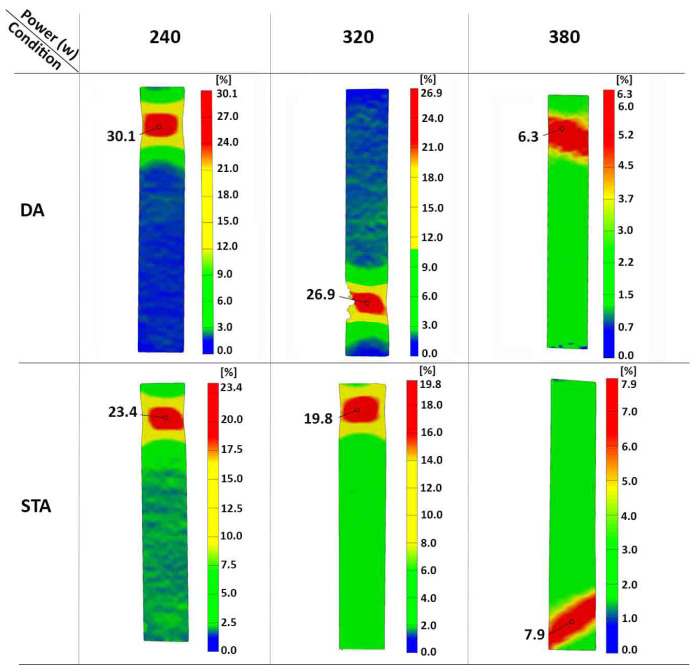
Distribution of the surface strains just before fracture for the DA and STA 300 MS processed by ASHM at 240 W, 320 W, and 380 W laser powers (one sample is presented per condition for better visualization).

The global fracture strains (obtained from extensometer measurements), presented in Table 5, showed that although the 320 W and 380 W samples in the DA condition were comparable to those in the STA condition, the 240 W sample exhibited a higher value of 5.9% (DA-240 W), relative to 4.4% for STA-240 W, at 95% CI. A more detailed examination of the strain distribution during tensile loading was undertaken using the digital image correlation technique. For all the DA and STA samples, the distribution of the surface strains remained nearly uniform during the elastic and uniform plastic deformation stages. Subsequently, deformation in the samples was localized, and the magnitude of surface strains varied. Figure 13 shows the distribution of the surface strains during the local deformation stage, just before fracture of the DA and STA samples. Overall, the strain distribution on the surface of the samples exhibited significant heterogeneity, which was highest for the DA-240 W sample. Table 5 shows the maximum surface strains at the local deformation area just before fracture—hereinafter referred to as local strains—for the DA and STA samples. The highest and lowest values of the local strains just before fracture were observed, respectively, for the 240 W and 380 W samples in the DA condition. By contrast, the local strains at fracture for the 240 W and 320 W samples in the STA condition were lower, and somewhat comparable to the 380 W sample, considering the standard deviations.

Overall, these results established important links between the processing (additive plus heat treatment), microstructure (retained austenite), and properties (global and local) that provide an important guide to achieving a number of strength-to-ductility combinations in support of diversifying 300 MS applications in industry. Where strength is paramount, STA of 300 MS may prove necessary, but the DA heat treatment provides a more effective option for achieving a greater strength–ductility balance. This was especially evident in the DA-240 W sample, which exhibited a mechanical response with the highest global and local strains and only a marginal strength reduction relative to the STA condition.

**Figure 14:**
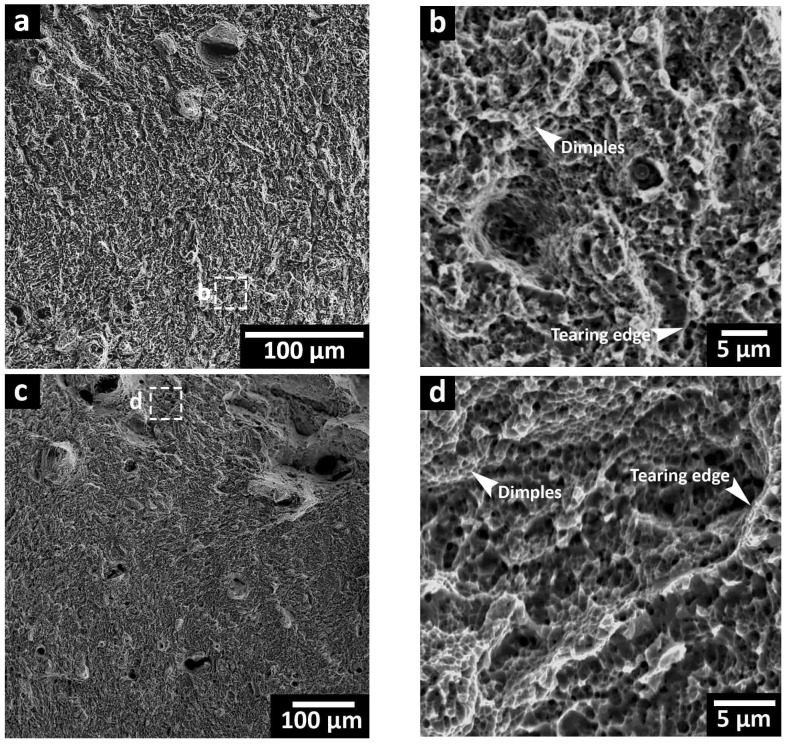
SEM fracture surfaces of: (**a**,**b**) STA-240 W and (**c**,**d**) DA-240 W.

Figure 14a–d show the fracture surface of STA-240 W and DA-240 W samples. Considering the similar fracture surface regardless of the laser power, only the heat-treated 240 W samples are presented. Low-magnification images of both STA and DA fracture surfaces showed a flat fracture surface without exhibiting a necking feature, as indicated in Figure 14a,c. The higher-magnification image of the STA fracture surface showed shallow dimples in limited areas of the surface, as indicated in Figure 14b. In addition, signs of a brittle failure can be seen in the form of planar cleavage with tearing edges, as indicated in Figure 14b. This confirmed a quasi-cleavage failure, which is commonly reported for heat-treated 300 MS [11,31,48]. Same features could be observed in the DA sample with more prominent dimples on the fracture surface compared to the STA sample. This indicates more localized plastic deformation resulting from the interaction of the advancing microcrack, with small packets of the reverted austenite available in the DA-240 W sample.

## 4. Discussion and Future Work

### 4.1. Microstructural Evolution

The ST of 300 MS produced by LPBF is reported to result in pore growth by merging the micropores during the grain growth process, which facilitates the motion of smaller pores [49]. By contrast, the aging process yields pore shrinkage or elimination due to precipitate formation, which distorts the grains and forces surrounding pores to shrink or break. Therefore, it is expected that the STA process will yield insignificant porosity changes, where the increased porosity content by the ST will be alleviated by the subsequent aging, which can be seen in Figure 5a,b. By contrast, the DA process is expected to result in a reduced porosity content by the precipitation of Ni_3_(Ti, Mo, Al). This is aligned with Figure 5c,d, where the percent porosity reduced from 0.016% in AB-240W2 to 0.01% in DA-240W2. However, considering the extremely minor porosity content in the AB state, as revealed by the µCT data (0.001–0.016%), the subsequent STA and DA treatments showed insignificant changes in porosity levels, regardless of the laser power (applied in this study), as shown in Table 3. It is worth mentioning that the measured porosity content may be marginally underestimated due to the image quality, voxel size, and filtration technique, where pores below an equivalent diameter of 5 µm were undetectable by the µCT measurements in this study.

All LPBF characteristics, including scan tracks, melt pool boundaries, and cellular solidification structures, were observed in the microstructure of 300 MS produced by the ASHM process. Post-ASHM heat treatment significantly influences the resulting microstructure of 300 MS, as observed in the present study. The elevated temperatures of the ST can achieve full recovery from the cellular solidification, with complete disappearance of the cellular structures [16,17,18]. The ST temperature and/or time are the key factors for the full dissolution of the segregated elements in the cellular spacing. Previous studies have shown an increased Mo and Ti content in the cellular spacing in the AB conditions [31]. The ST at 825 °C for 1 h selected for the present study showed the removal of the melt pool boundaries and partial disappearance of the cellular boundaries upon STA, regardless of the laser power, as shown in Figure 8a–c. Therefore, a higher ST temperature could be used for full homogenization of LPBF characteristics. This homogenization may result in reduced anisotropy of the mechanical properties compared to the AB conditions [7], but any concomitant grain growth can lower the YS, UTS, and elongation [5].

After ASHM, the DA heat treatment also significantly influenced the resulting microstructure of 300 MS. As shown in Figure 9, DA at 490 °C for 6 h partially retained the remnants of the AB cellular solidification structure, where the cell boundaries changed from their continuous nature (in the AB state) into fragmented, discontinuous traces after DA. The diffusion of the segregated Mo and Ti along the cell boundaries needs much higher energy than that provided by DA, which explains the presence of the cell boundaries in the DA state [14,19]. The fragmentation of the cell boundaries has been reported to be due to the intermetallic precipitation of Ni_3_(Ti, Mo, Al), which consumes the segregated Mo and Ti within the cell boundaries and forms discontinuous cell boundaries [3]. The complete dissolution of the cellular structure was reported to be impossible to achieve in the DA process, even if the aging temperature was increased up to 560 °C and the holding time was raised to 12 h [31].

The ASHM process resulted in retained austenite in 300 MS, which is similar to the observations for 300 MS processed in standalone LPBF systems. This is attributed to the similar thermal cycling (i.e., cyclic heating and cooling among the built layers) and solidification conditions. Specifically, upon melting a new powder layer, previously solidified layers were austenitized and rapidly cooled again. The constant heat flow from molten regions to the build platform inhibited these layers from achieving the martensite final temperature (M_f_), leaving some austenite retained in the microstructure. This small fraction of retained austenite was stabilized at room temperature by micro-segregation of Ni, since it is known as an austenite stabilizer [19]. This phenomenon has also been reported for other martensitic steels produced by LPBF [50,51,52]. In the present study, the amount of retained austenite, presented in Figure 10d, varied from 4.9 to 9.3% in the AB condition for the 240 W and 380 W samples, respectively. It is believed that a higher laser power, while keeping other process parameters constant, increases the heat input; thus, the heat flow is higher from the molten regions to the underlying layers. Consequently, the temperature of these solidified layers will be higher than the case for a lower laser power, resulting in a higher amount of retained austenite.

Austenite reversion is commonly reported for 300 MS upon aging [1,11,14,20,47]. The reported Ni micro-segregation in the AB conditions of LPBF 300 MS results in the formation of reverted austenite at the martensite lath boundaries [53]. This explains the lower fraction of reverted austenite in the STA state compared to the DA condition, where the ST homogenized the material before aging and reduced Ni segregation. The subsequent aging process after ST did not significantly induce reverted austenite (STA), where a maximum of 3% reverted austenite could be observed for the STA-380 W sample. Byn contrast, the DA samples exhibited up to 13.4% austenite for the DA-240 W sample. This austenite reversion occurred at an aging temperature of 490 °C, which is far below the austenite start temperature (A_s_) of ~600 °C due to austenite stabilization by Ni [14].

### 4.2. Mechanical Properties

STA and DA samples achieved a dramatic increase in hardness and strength upon aging. The theoretical YS of the aged material ($\sigma_{a}$) can be described as the combination of the following strengthening factors, expressed as:

(1) $$\sigma_{a}=\sigma_{m}+\sigma_{ss}+\sigma_{gs}+\sigma_{d}+\sigma_{p}\;$$

where $\sigma_{m}$ is the matrix strength, $\sigma_{ss}$ is the solid-solution strengthening, $\sigma_{gs}$ is the grain size strengthening, $\sigma_{d}$ is the dislocation strengthening, and $\sigma_{p}$ is the precipitation hardening. Equation (1) can be rewritten as a function of the YS before aging ($\sigma_{0}$), as follows:

(2) $$\sigma_{a}=\sigma_{0}+\Delta \sigma_{ss}+\Delta \sigma_{gs}+\Delta \sigma_{d}+\sigma_{p}\;$$

where $\Delta \sigma_{ss}$ is the change in the solid-solution strengthening that solely transpires from the change in the concentration of solute atoms during the aging process, $\Delta \sigma_{gs}$ is the change in grain size strengthening upon aging, and $\Delta \sigma_{d}$ is the change in the dislocation strengthening due to aging.

As mentioned, the change in the solid-solution strengthening ($\Delta \sigma_{ss}$) depends on the change in the concentration of the solute atoms in the matrix due to precipitation hardening effects during the aging process. Paul et al. [7] showed that the concentration of solute elements, such as Ni, Ti, and Mo, decrease in the matrix by 45, 15, and 10%, respectively. In the present study, the minor concentrations of Ti (0.96 at.%) and Mo (3 at.%) in the 300 MS composition reduced their respective contributions towards solid-solution strengthening. Therefore, this calculation can mainly be based on the effect of the change in the Ni concentration. In this case, the change in solid-solution strengthening can be estimated using the Labusch model, as follows [54]:

(3) $$\Delta \sigma_{ss}=3\;k\;\left(C_{a}^{2/3}-C_{0}^{2/3}\right)\;$$

where *k* is the solid solution hardening (SSH) coefficient—obtained by fitting empirical data to the Labusch expression—and is constant for a specific solvent–solute combination. Kadambi et al. reported a *k* value equal to 40 MPa/at.%^2/3^ for the Fe-Ni binary alloy [55]. $C_{a}$ and $C_{0}$ are the concentrations of the Ni solute atoms in the matrix after and before aging, respectively. Considering the 45% reduction in the Ni concentration upon aging, $C_{a}\;$= 9.68 at.% and $C_{0}\;$= 17.6 at.%. Therefore, $\Delta \sigma_{ss}\;$= −267 MPa, which indicates a reduction in the YS upon aging as a consequence of the lower concentration of Ni solute atoms in the Fe matrix.

The change in grain size upon aging ($\Delta \sigma_{gs}$) was negligible and supported by the findings of Mutua et al. of a statistically similar grain size in the AB, DA, and STA conditions for LPBF-processed 300 MS using the same ASHM technology [16] as in the present study. Although the semi-coherent precipitates that form during aging will cause minor distortion and increase the number of dislocations in the matrix [56], this term ($\Delta \sigma_{d})$ has not been considered in our theoretical calculations.

The material is mainly strengthened by the homogeneous distribution of intermetallic nanoprecipitates of Ni_3_(Ti, Mo, Al) in the martensite matrix following the Orowan strengthening mechanism, expressed as [14]:

(4) $$\sigma_{p}=\frac{Gb}{2\pi k\left(\lambda -d\right)}ln\frac{\left(\lambda -d\right)}{2b}\;$$

(5) $$\frac{1}{k}=\frac{1}{2}\left(\frac{1}{1-\upsilon }+1\right)\;$$

where G is the shear modulus of the matrix, b is the Burgers vector, λ is the interspace of the precipitates, d is the equivalent spherical diameter of the precipitates, and υ is the Poisson’s ratio. G is 73.26 GPa [7], b is 0.249 nm [47] for 300 MS, and υ is 0.3. λ and d were reported by Tan et al. to be 25 and 14 nm, respectively [56]. Similar precipitate sizes and spacings were thought to transpire in the present study due to the similarity in the starting microstructure and aging treatment (490 °C for 6 h). Thus, inserting these values into Equation (4) yielded $\sigma_{p}$ equal to 992 MPa.

It is noteworthy that Equation (4) is commonly used for ideally spherical precipitates. As presented in [56], the precipitates are needle-shaped with a diameter of 8 nm and a length of 30 nm. To consider the morphology (aspect ratio) of the precipitate particles, where the length is larger than the diameter, the modified equation by Sonderegger et al. [57] can be applied, as follows [58]:

(6) $$\phi =h^{1/6}{\left(\frac{2+h^{2}}{3}\right)}^{-1/4}\;$$

(7) $$\sigma_{p-modified}=\frac{\sigma_{p}}{\phi }\;$$

where ϕ is the correction shape factor and *h*—the particle aspect ratio—equals the length/diameter of the precipitate. Substituting into Equations (6) and (7), ϕ = 0.82 and $\sigma_{p-modified}$ = 1211 MPa.

Now, the contributing factors in Equation (2) have been defined. Since $\sigma_{0}$ is the YS before aging, the YS of the STA samples can be calculated based on the YS of the STed samples when the matrix is homogenized and contains no retained austenite or cellular structures. The YS of STed samples can be estimated by Tabor’s relationship ($\sigma_{0}\approx 3HV$ [59]) to be 1011 MPa using Figure 11b (i.e., for the STed 240 W sample). The calculated YS $\left(\sigma_{a}\right)$ of the STA-240 W sample will be 1955 MPa. This predicted value is comparable to the measured YS of 2030 ± 9 MPa for the STA-240 W sample with a ~4% estimation error. These calculations also agree well with the other STA samples (i.e., STA-320 W and STA-380 W), which emphasizes that precipitation hardening is the primary strengthening mechanism for the STA samples and that the impact of solid-solution strengthening upon aging is minor.

To apply Equation (2) to the DA samples, $\sigma_{0}$ is the YS of the AB samples, which can be estimated using Tabor’s relationship [59]. For instance, the YS of AB-240 W is equal to 3 × 380 HV = 1140 MPa. Then, the theoretical YS of DA-240 W is equal to 2084 MPa. The theoretical YS using Equation (2) could not be directly applied for DA materials, where only the martensite phase is considered in the calculations, and the austenite content is neglected. This can lead to significant error, specifically when the amount of reverted austenite cannot be neglected, as under the DA conditions. Recent studies have suggested the employment of the rule of mixtures to account for the reverted austenite contribution towards the YS reduction, as given in Equation (8) [60,61]:

(8) $$\sigma_{y}=\sigma_{y}^{\alpha }\;\left(1-V_{\gamma }\right)+\sigma_{y}^{\gamma }\;V_{\gamma }\;$$

where $\sigma_{y}$ is the theoretical YS of the DA materials with reverted austenite, $\sigma_{y}^{\alpha }$ is the YS of the DA martensitic matrix, $\sigma_{y}^{\gamma }$ is the YS of reverted austenite, and $V_{\gamma }$ is the austenite volume fraction. Applying Equation (8) to the DA-240 W sample—where $\sigma_{y}^{\alpha }$ equals 2084 MPa, $\sigma_{y}^{\gamma }$ equals 217 MPa [61], and $V_{\gamma }$ equals 13.4% (Figure 10d)—yields a theoretical $\sigma_{y}$ for DA-240 W of 1834 MPa. This estimated value matches well with the measured value of 1884 ± 31 MPa for DA-240 W (Table 5), with a ~3% estimation error. Considering the $V_{\gamma }$ of 8% for DA-320 W and 11.7% for DA-380 W, the calculated YS of DA-320 W and DA-380 W will be 1934 and 1876 MPa, respectively. This estimation closely matches their measured values (from Table 5) of 1895 ± 23 and 1860 ± 3 MPa, respectively, with ~2% error for DA-320 W and ~1% error for DA-380 W. This emphasizes the good prediction accuracy of the YS of DA samples using the linear rule of mixtures approach.

Although the reverted austenite in the DA samples marginally reduced the YS and UTS by ~7% compared to the STA conditions, it contributed to enhanced ductility in the DA samples by up to ~34% compared to the STA conditions. In earlier work, the enhanced ductility has been attributed to transformation-induced plasticity (TRIP) effects [62]. For instance, DA-240 W featured up to 13.4% reverted austenite, which promoted a more stable plastic deformation, particularly after necking, and up to ~5.9% fracture strain (global), compared to only 4.4% globally for STA-240 W. This mechanism has been discussed in detail by Wang et al., where the reverted austenite promoted stable plastic deformation in a two-stage process [4]. At early deformation stages, the plastic deformation was assisted by the reverted austenite. As the plastic deformation progressed, the freshly transformed martensite through TRIP effects enhanced the material’s load-bearing capacity, and since the new martensite featured less defects and precipitates, it could promote stable plastic deformation. This mechanical response can be effective when the reverted austenite is sufficient to trigger this mechanism, as was the case for the DA-240 W sample with 13.4% reverted austenite, which was the highest content among the DA samples and resulted in enhanced global and local strains, as shown in Table 5 and Figure 13. However, the 8% reverted austenite in DA-320 W did not significantly trigger the presented mechanism or improve the material ductility compared to STA-320 W. For this reason, recent studies are concerned with increasing the reverted austenite in precipitation-hardened 300 MS to enhance its ductility [7,20,53,63]. For instance, Paul et al. [7] thermally cycled 300 MS twice at 750 °C for 5 min, then aged it at 480 °C for 6 h, which led to ~12% reverted austenite and 5.3 ± 0.3% fracture strain. The authors reported additional gains in the fracture toughness associated with the increased reverted austenite by ~50% compared to the STA conditions [7].

To sum up, the gains of the STA treatment were the homogenization of the AB microstructure, which could marginally reduce the anisotropy of the material and lead to the formation of a fully martensitic microstructure with peak YS and UTS. However, the DA treatment resulted in an increased reverted austenite content, which can be advantageous to enhance the ductility by up to ~34%, with a minor reduction in YS by ~7% compared to STA conditions. In addition, the DA treatment reduced the cost of an additional and seemingly ineffective heat treatment cycle, as well as the possible risks of dimensional instabilities associated with the high temperature of ST. Therefore, based on the present work, it is recommended to directly age 300 MS produced by ASHM. However, the conditions of the DA treatment should be selected to achieve ~13% reverted austenite, such that gains of ductility can be triggered by TRIP effects, which explains the contradiction among the results reported in the literature on the efficiency of the DA treatment compared to STA.

## 5. Conclusions

The present work investigated the combined effect of heat treatment and laser power on the microstructure and mechanical properties of 18Ni-300 maraging steel (300 MS) processed by additive–subtractive hybrid manufacturing (ASHM). The effectiveness of two heat treatment cycles: solution treatment + aging (STA) and direct aging (DA), was evaluated for 300 MS produced by ASHM at three laser powers (240 W, 320 W, and 380 W). The following main conclusions can be drawn from this study:

1. Neither the STA nor DA heat treatments had any significant effect on the porosity of 300 MS produced by ASHM at the different laser powers studied.
2. DA at 490 °C for 6 h yielded a similar microstructure to the as-built (AB) samples, with partial dissolution of the cellular substructures into shorter fragments due to the precipitation of Ni intermetallic compounds. ST at 825 °C for 1 h was insufficient for full microstructural homogenization; thus, 300 MS in the STA condition still featured minor traces of cellular substructures within the matrix.
3. Compared to the AB samples, DA increased the austenite content up to 13.4% due to the reversion of martensite into austenite, stabilized by Ni micro-segregation in the AB conditions, while ST and STA samples exhibited almost fully martensitic microstructures.
4. The macro- and micro-hardness responses of STA (54 HRC and 618 HV_0.5_) and DA (54 HRC and 606 HV_0.5_) samples were comparable, where the low fraction of reverted austenite in DA samples did not significantly influence the hardness measurements.
5. The reverted austenite in DA samples marginally reduced the YS and UTS by ~7% compared to the STA samples, while enhancing the ductility by up to ~34% through transformation-induced plasticity (TRIP) effects. This ductility enhancement could be triggered at the highest reverted austenite content of ~13.4% in the present study.
6. The microstructural and mechanical performance of heat-treated 300 MS produced by ASHM was found to be comparable to those produced by stand-alone additive manufacturing methods (e.g., laser powder bed fusion). This can promote wider applications of 300 MS with ASHM for producing intricate geometries with complex inner features and a high-precision machined surface quality.

## Figures and Tables

**Table 2:** Chemical composition of 300 MS powder (balance Fe).

Elements	Ni	Co	Mo	Ti	Cr	Al	Si	Mn	C	P	S	O	N
Composition (wt.%)	17.9	8.9	5	0.8	0.2	0.11	0.08	0.06	0.01	0.008	0.006	0.04 ppm	0.02 ppm

**Table 3:** Sample conditions and designations used in the current study.

Condition	Laser Power (W)	Sample Designation
As-built	240	AB-240 W
	320	AB-320 W
	380	AB-380 W
Solution-treated	240	ST-240 W
	320	ST-320 W
	380	ST-380 W
Solution-treated + aged	240	STA-240 W
	320	STA-320 W
	380	STA-380 W
Direct aging	240	DA-240 W
	320	DA-320 W
	380	DA-380 W

**Table 4:** X-ray µCT scan results before and after the different heat treatment conditions.

Laser Power	Before Heat Treatment	Porosity %	Largest Pore Diameter (µm)	Number of Pores	After Heat Treatment	Porosity %	Largest Pore Diameter (µm)	Number of Pores
240 W	AB-240W1	0.003	86	569	STA-240W1	0.003	84	552
	AB-240W2	0.001	40	472	DA-240W2	0.001	40	441
320 W	AB-320W1	0.005	81	386	STA-320W1	0.005	63	425
	AB-320W2	0.016	99	1774	DA-320W2	0.01	92	1138
380 W	AB-380W1	0.001	85	132	STA-380W1	0.0003	79	41
	AB-380W2	0.001	45	242	DA-380W2	0.0003	41	91

**Table 5:** Average tensile properties of as-built (AB) and heat-treated (STA and DA) 300 MS built by ASHM at different laser powers.

Sample	YS (MPa)	STD	UTS (MPa)	STD	E (GPa)	STD	Fracture Strain (%)		STD	Ref.
							Local	Global		
AB-240 W	1006	15	1158	3	169	5	-	12.8	0.7	Previous study by current authors [27]
AB-320 W	1062	14	1171	5	170	3	-	12.9	0.4	
AB-380 W	1022	19	1160	11	161	1	-	12.5	0.8	
STA-240 W	2030	9	2080	24	185	9	23.4	4.4	0.1	Current study
STA-320 W	1983	17	2066	6	182	2	19.8	4.3	0.2	
STA-380 W	1973	38	2073	10	192	7	7.9	3.5	0.7	
DA-240 W	1884	31	1950	14	164	3	30.1	5.9	0.5	
DA-320 W	1895	23	1943	14	173	8	26.9	4.2	0.1	
DA-380 W	1860	3	1923	7	165	4	6.3	3.2	1	

## Data Availability

The authors confirm that the data supporting the findings of this study are available within the article.
